# Induction of Connective Tissue Growth Factor Expression by Hypoxia in Human Lung Fibroblasts via the MEKK1/MEK1/ERK1/GLI-1/GLI-2 and AP-1 Pathways

**DOI:** 10.1371/journal.pone.0160593

**Published:** 2016-08-03

**Authors:** Yi Cheng, Chien-huang Lin, Jing-Yun Chen, Chien-Hua Li, Yu-Tin Liu, Bing-Chang Chen

**Affiliations:** 1 School of Respiratory Therapy, College of Medicine, Taipei Medical University, Taipei, Taiwan; 2 Gradual Institute of Medical Sciences, College of Medicine, Taipei Medical University, Taipei, Taiwan; 3 School of Pharmacy, College of Pharmacy, Taipei Medical University, Taipei, Taiwan; 4 Department of Internal Medicine, School of Medicine, College of Medicine, Taipei Medical University, Taipei, Taiwan; Medical University of South Carolina, UNITED STATES

## Abstract

Several reports have indicated that hypoxia, GLI, and connective tissue growth factor (CTGF) contribute to pulmonary fibrosis in idiopathic pulmonary fibrosis. We investigated the participation of mitogen-activated protein kinase kinase (MEK) kinase 1 (MEKK1)/MEK1/ERK1/GLI-1/2 and activator protein-1 (AP-1) signaling in hypoxia-induced CTGF expression in human lung fibroblasts. Hypoxia time-dependently increased CTGF expression, which was attenuated by the small interfering RNA (siRNA) of GLI-1 (GLI-1 siRNA) and GLI-2 (GLI-2 siRNA) in both human lung fibroblast cell line (WI-38) and primary human lung fibroblasts (NHLFs). Moreover, GLI-1 siRNA and GLI-2 siRNA attenuated hypoxia-induced CTGF-luciferase activity, and the treatment of cells with hypoxia induced GLI-1 and GLI-2 translocation. Furthermore, hypoxia-induced CTGF expression was reduced by an MEK inhibitor (PD98059), MEK1 siRNA, ERK inhibitor (U0126), ERK1 siRNA, and MEKK1 siRNA. Both PD98059 and U0126 significantly attenuated hypoxia-induced CTGF-luciferase activity. Hypoxia time-dependently increased MEKK1, ERK, and p38 MAPK phosphorylation. Moreover, SB203580 (a p38 MAPK inhibitor) also apparently inhibited hypoxia-induced CTGF expression. The treatment of cells with hypoxia induced ERK, GLI-1, or GLI-2 complex formation. Hypoxia-induced GLI-1 and GLI-2 translocation into the nucleus was significantly attenuated by U0126. In addition, hypoxia-induced ERK Tyr204 phosphorylation was impeded by MEKK1 siRNA. Moreover, hypoxia-induced CTGF-luciferase activity was attenuated by cells transfected with AP-1 site mutation in a CTGF construct. Exposure to hypoxia caused a time-dependent phosphorylation of c-Jun, but not of c-Fos. Chromatin immunoprecipitation (ChIP) revealed that hypoxia induced the recruitment of c-Jun, GLI-1, and GLI-2 to the AP-1 promoter region of CTGF. Hypoxia-treated cells exhibited an increase in α-smooth muscle actin (α-SMA) and collagen production, which was blocked by GLI-1 siRNA and GLI-2 siRNA. Overall, these data implied that the MEKK1/MEK1/ERK1/GLI-1/GLI-2, and AP-1 pathways mediated hypoxia-induced CTGF expression in human lung fibroblasts. Furthermore, GLI-1 and GLI-2 found to be involved in hypoxia-induced α-SMA and collagen expression.

## Introduction

Idiopathic pulmonary fibrosis (IPF) is the most common and lethal form of all interstitial lung diseases (ILDs), with an estimated 5-year survival rate for approximately 20% for affected patients. Patients with IPF have a low quality of life because of dyspnea, chest tightness, and severe dry cough [1–4]. Among all patients who undergo bilateral lung transplantation, ILD patients are ranked third in number [5]. Numerous studies have reported that lung fibroblast overdivision and extracellular matrix (ECM) accumulation and deposition are the stages of disease progression [4]. The pathophysiology of IPF remains unclear, and current treatment can provide only supportive care to patients with IPF [1, 4]. Increased levels of hypoxia are followed by IPF progression, thus exacerbating the symptoms of these patients. Moreover, hypoxia stimulates lung fibroblasts to undergo proliferation, accumulation, and differentiation [6]. In trauma lesions in the lungs, residual fibroblasts are the controller cells of ECM deposition and connective tissue growth factor (CTGF) expression [7]. Wang et al. (2009) found that CTGF overexpression induced fibroblast differentiation and that hypoxia mediated fibrosis development [8]. Thus, CTGF may play a major role in hypoxia-induced pulmonary fibrosis.

The hedgehog signaling pathway is highly regulated because it plays a crucial role in embryonic development, tissue patterning, and organogenesis, whereas GLI proteins are the downstream transcriptional factors of this pathway [9, 10]. Hedgehog signaling responses are mediated by PTCH1, a 12-pass integral membrane protein, and Smoothened, a 7-pass integral membrane protein [11]. In addition, a “noncanonical pathway” regulates hedgehog signaling as well as the activity of GLI proteins, including the ERK, PI3K/Akt, and GPCR-PLC-c-jun pathways [12]. The hedgehog pathway plays a major role in IPF pathogenesis. Sonic hedgehog (SHH) and PTCH1 as well as GLI-1 and GLI-2 are highly expressed in the lung tissue and fibroblasts of patients with IPF [13]. In addition, blocking hedgehog pathway signaling through SHH or PTCH1 or directly knocking down GLI-1 and GlLI-2 proteins apparently lowered the level of bleomycin-induced pulmonary fibrosis in mice [14]. However, the roles of GLI-1 and GLI-2 in regulating CTGF expression in lung fibroblasts through hypoxia remain unexplored.

CTGF, a CCN family member, is well known to be a crucial mediator in ILDs, including pulmonary fibrosis [15]. In the resting stage of fibroblasts, CTGF is expressed at extremely low concentrations, but it is overexpressed at an extremely high level by specific stimuli (e.g., hypoxia or TGF-β) [8, 16]. Numerous studies have attributed increased CTGF production to stress fiber production, ECM protein accumulation, and myofibroblast differentiation [16–18]. Thus, these studies have concluded that in interstitial pulmonary fibrosis, CTGF is a key mediator contributing to disease progression. Several reports have indicated that the human CTGF promoter contains several transcription factor binding sites, including those for nuclear factor-κB (NF-κB), Ets-1, signal transducer and activator of transcription (STAT), and AP-1 [19–21]. Yu et al. (2009) found that AP-1 stimulation contributed to thrombin-induced CTGF expression [22]. Nonetheless, the mechanism through which AP-1 mediates hypoxia-induced CTGF expression has yet to be identified.

Mitogen-activated protein kinase kinase kinase 1 (MEKK1) and ERK regulate chemotaxis, immunocyte recruitment, and inflammatory protein production, in addition to participating in the noncanonical regulation of GLI-1 and GLI-2 proteins [12]. Studies have shown that MEK stabilizes GLI proteins, and typically enhances the transcriptional activity of GLI-1. Moreover, GLI-1 has been demonstrated to be a novel substrate of ERK [10, 23–25]. In addition, ERK activation was found to be a key step in GLI activation, which in turn mediated transcription factor activity that activated profibrotic genes thereby contributing to interstitial pulmonary fibrosis [26]. For instance, ERK is involved in matrix metalloproteinase-9 expression and interstitial pulmonary fibrosis [27]. Aberger et al. (2012) reported that ERK regulated GLI protein activation via the Ras-MEK-ERK pathway in malignant cells [12]. Nonetheless, the roles of MEKK1/MEK1/ERK1/GLI, and AP-1 in hypoxia-mediated CTGF expression remain unclear. In the current study, we concluded that hypoxia-induced CTGF expression through MEKK1/MEK1/ERK1 activation and recruited c-Jun, GLI-1, and GLI-2 to the AP-1 binding site of the CTGF promoter region in human lung fibroblasts. Moreover, GLI-1 and GLI-2 mediated the induction of hypoxia-induced α-smooth muscle actin (α-SMA) and collagen expression.

## Materials and Methods

### Materials

A human lung fibroblast cell line (WI-38) was purchased from American Type Culture Collection (Manassas, VA, USA). The primary normal human lung fibroblasts (NHLFs) were purchased from Lonza (Walkers-ville, MD, USA). U0126, PD98059, and SB203580 were obtained from Calbiochem-Novabiochem (San Diego, CA, USA). Lipofectamine 3000 and Lipofectamine Plus reagent, and a minimum essential medium were obtained from Invitrogen Life Technologies (Carlsbad, CA, USA). The CTGF ELISA kit was purchased from Assay BioTech (Sunnyvale, CA, USA). An α-tubulin antibody was acquired from Transduction Laboratories (Lexington, KY, USA). A GLI-1 antibody was obtained from R&D systems (Minneapolis, MN, USA). The siRNAs of MEK1 and ERK1, as well as antibodies specific for the ERK Tyr204 phosphorylation site, ERK; the c-Fos Ser374 phosphorylation site, c-Fos; the c-Jun Ser63 phosphorylation site, c-Jun; the p38 Tyr182 phosphorylation site, p38α; CTGF; and rabbit polyclonal IgG, as well as antirabbit, antimouse, and antigoat IgG-conjugated horseradish peroxidase were acquired from Santa Cruz Biotechnology (Dallas, TX, USA). GLI-1 siRNA, a mixture containing 2 specific GLI-1 siRNAs, was used with the following sequence: 5’-CUA CUG AUA CUC UGG GAU A-3’ (sense1) and 5’-GUC AUA CUC ACG CCU CGA A-3’ (sense2), in addition, GLI-2 siRNA, a mixture containing 2 specific GLI-2 siRNAs, was used with the following sequence: 5’-GAC AUG AGC UCC AUG CUC A-3’ (sense1) and 5’-CGA UUG ACA UGC GAC ACC A-3’ (sense2), as well as MEKK1 siRNA, a mixture containing 2 specific MEKK1 siRNAs, was used with the following sequence: 5’-CAA AGG UGC CAA UUU GCU A -3’ (sense1) and 5’-GGA AUU UCC UGC UGA AUU U -3’ (sense2), which were synthesized by Sigma (St. Louis, MO, USA). Antibodies specific for GLI-2 and α-SMA were obtained from Abcam (Cambridge, MA, USA). An antibody specific for MEK1 was purchased from GeneTex (Irvine, CA, USA). A Collagen type 1 antibody was purchased from Rockland (Limerick, PA, USA). All materials for western blotting were acquired from Bio-Rad (Hercules, CA, USA). The CTGF promoter (-747/+214) luciferase plasmid was gifted by Dr. M.-L. Kuo (National Taiwan University, Taipei, Taiwan), pcDNA was gifted by Dr. M.-C. Chen (Taipei Medical University, Taipei, Taiwan), and pBK-CMV-Lac Z was gifted by Dr. W-W. Lin (National Taiwan University, Taipei, Taiwan).

### Cell Culture

A human lung fibroblast (WI-38) cell culture was prepared as described previously [28]. In brief, WI-38 cells were cultured in a growth medium. NHLFs were cultured in a fibroblast growth medium (FGM), with growth supplements provided in the manufacturer’s FGM singleQuot kit (Lonza, Verviers, Belgium). After confluence, the cells were passaged onto 35-mm dishes for CTGF ELISA; onto 60-mm dishes for transfection, western blotting, coimmunoprecipitation; onto 100-mm dishes for chromatin immunoprecipitation (ChIP) and nuclear protein extraction; onto 4-well culture slides for confocal microscopy; and onto 12-well plates for examination of the luciferase activity. For hypoxia treatment, the cells were subjected to 1% O_2_ hypoxia, which was achieved using the ProOx model 110 chamber at 37°C, flushed with a gas mixture of 5% CO_2_/95% N_2_, purchased from BioSpherix (New York, NY, USA). The oxygen concentration within the chamber was maintained at 1% by using a ProOx 110 oxygen regulator (BioSpherix).

### CTGF Supernatant Measurement

The WI-38 cells were subjected to 1% O_2_ hypoxia for 0–48 h. Afterward, the cell starvation medium was collected for further ELISA. Secreted CTGF was detected using the ELISA kit as instructed by the manufacturer.

### SiRNA Transfection

The WI-38 or NHLF cells (2 × 10^5^ cells/well) were transfected for 24 h with control siRNA, GLI-1 siRNA, GLI-2 siRNA, MEK1 siRNA, and ERK1 siRNA by using lipofetamine 3000 reagents in accordance with the manufacturer’s instructions. After siRNA transfection, the cells were subjected to hypoxia (1% O_2_) for the indicated time intervals for western blotting or luciferase activity assays.

### Luciferase Activity

The CTGF-luciferase activity assay was previously described [28]. In brief, the cells were transfected with CTGF-Luc (-747/+214), AP-1-mt-CTGF-Luc, and Lac Z for 6 h, after which they were cultured overnight in a basal medium without fetal bovine serum. The cells were subjected to hypoxia (1% O_2_) for an additional 24 h, and then the luciferase assay system was used (Promega, Madison, WI, USA) to assess luciferase activity. The ratio of cells with and without stimulation was referenced to measure the level of luciferase activity induction.

### Western Blotting

The WI-38 cells were pretreated with a vehicle (DMSO), PD98059, or U0126 for 20 min, or transfected with GLI-1 siRNA, GLI-2 siRNA, MEK1 siRNA, or ERK1 siRNA for 24 h before they were subjected to hypoxia. Immunoreactivity was detected using western blotting analysis, as previously described [28].

### Nuclear Protein Extraction

The WI-38 cells were pretreated with a vehicle (DMSO) or U0126 for 20 min in 100-mm dishes before they were stimulated with hypoxia for an additional 2 h. The cell lysates were obtained with a cytoplasmic and nuclear protein extraction kit purchased from TOOLS (New Taipei City, Taiwan), which was used in accordance with the manufacturer’s instruction. Immunoreactivity was detected using western blotting analysis.

### Coimmunoprecipitation Assay

The WI-38 cells were incubated in 60-mm dishes before they were subjected to hypoxia (1% O_2_) for 10 min. The cell lysates were harvested as previously described [29]. Afterward, the cell lysates were immunoprecipitated with an antibody against ERK in the presence of mag sepharose magnetic beads. After elution from the beads, the cell lysates were immunodetected using western blotting. The ERK was detected as the endogenous control.

### Chromatin Immunoprecipitation Assay

The WI-38 cells were subjected to hypoxia (1% O_2_) for the indicated time before they were fixed with formaldehyde for an additional 10 min. The c-Jun, GLI-1, and GLI-2 binding to the AP-1 binding site of the CTGF promoter region was detected through a ChIP assay, performed as described previously [28]. The primer sequences used to perform polymerase chain reaction amplification on the CTGF promoter site were AP-1, sense: 5’-GGA TGT ATG TCA GTG GAC AGA-3’, and antisense: 5’-AAG CGC AGT ATT TCC AGC ACC-3’.

### Immunofluorescence Staining and Confocal Microscopy

The cells were subjected to hypoxia (1% O_2_) for 2 h. Nuclear GLI-1 and GLI-2 were detected using immunofluorescence staining and confocal microscopy, as described previously [28]. In brief, slides were blocked with 5% normal calf serum and incubated with antibodies specific to GLI-1 or GLI-2 overnight, after which they were incubated with a fluorescein isothiocyanate (FITC)-conjugated secondary antibody for an additional 1 h. The slides were counterstained with DAPI to visualize the nuclei. They were then examined under a confocal fluorescence microscope (Leica TCS SP5, Wetzlar, Germany).

### Statistical Analysis

All data passed the normality test. The results are presented as the mean ± standard error of the mean (SEM) or standard deviation (SD) based on at least 3 independent experiments. One-way analysis of variance (ANOVA) was performed followed by the Dunnett test, to analyze the differences between groups. A *p* value of <0.05 was considered statistically significant.

## Results

### Mediating Effect of GLI-1 and GLI-2 on Hypoxia-Induced CTGF Expression

**Fig 1:**
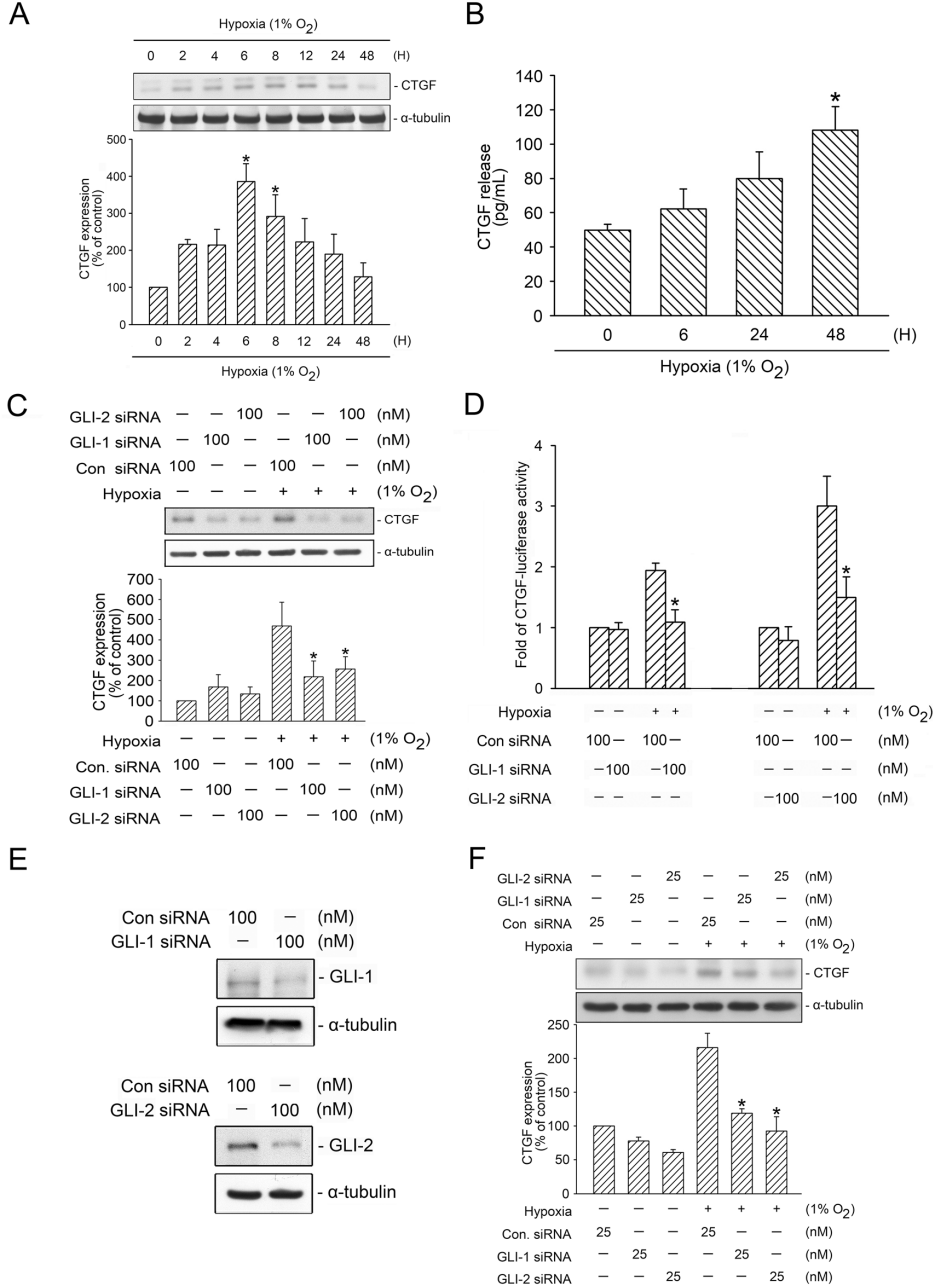
Mediation of GLI-1 and GLI-2 in hypoxia-induced CTGF expression. A, WI-38 cells were subjected to hypoxia (1% O_2_) from 0 to 48 h. CTGF and α-tubulin levels in cell lysates were detected using western blotting. The results are expressed as the mean ± SEM of three independent experiments. * *p* < 0.05, compared with the control group without hypoxia stimulation. B, WI-38 cells were subjected to hypoxia for 0–48 h. The medium CTGF level was detected with ELISA. The results are expressed as the mean ± SEM of four independent experiments. * *p* < 0.05, compared with the control group without hypoxia stimulation. C, WI-38 cells were transfected with control siRNA (con siRNA), GLI-1 siRNA, and GLI-2 siRNA. After 24 h, the cells were subjected to hypoxia for an additional 6 h. CTGF and α-tubulin levels were detected, as described previously. The results are expressed as the mean ± SEM of three independent experiments. * *p* < 0.05, compared with hypoxia plus the control siRNA group. D, WI-38 cells were transfected with control siRNA (con siRNA), GLI-1 siRNA, GLI-2 siRNA, 0.5 μg of CTGF-Luc, and 0.1 μg of pBK-CMV-Lac Z. After 24 h, the cells were subjected to hypoxia (1% O_2_) for an additional 24 h. The luciferase activity assay is described in the “Materials and Methods” section. The results are expressed as the mean ± SEM of three independent experiments performed in duplicate. * *p* < 0.05, compared with hypoxia plus the control siRNA group. E, WI-38 cells were transfected with control siRNA (con siRNA), GLI-1 siRNA, and GLI-2 siRNA. After 24 h, GLI-1, GLI-2, and α-tubulin levels were detected using western blotting. Typical traces represent three independent experiments with similar results. F, NHLF cells were transfected with control siRNA (con siRNA), GLI-1 siRNA, and GLI-2 siRNA for 24 h. The cells were subjected to hypoxia for an additional 6 h. CTGF and α-tubulin levels were detected, as described previously. The results are expressed as the mean ± SEM of three independent experiments. * *p* < 0.05, compared with hypoxia plus the control siRNA group.

Recent studies have shown that GLI-1 and GLI-2 play a major role in pulmonary fibrosis [13, 14, 30]. In addition, CTGF has been described as a crucial mediator in interstitial pulmonary fibrosis and is regulated by hypoxia [8, 16, 18, 20]. However, the roles of GLI-1 and GLI-2 in hypoxia-induced CTGF expression remain unknown. The present study examined whether GLI-1 and GLI-2 could mediate hypoxia-induced CTGF expression in human lung fibroblasts (WI-38 cells). Cells subjected to hypoxia (1% O_2_) time-dependently induced CTGF expression, with the peak affecting time at 6 h of hypoxia treatment (Fig 1A). Because CTGF is a secreted protein [31], we aimed to determine whether hypoxia can induce CTGF release into the medium. Fig 1B shows that cells stimulated with hypoxia (1% O_2_) for 0–48 h, exhibited an increase in hypoxia-induced release of CTGF into the medium in a time dependent manner. After 48 h of stimulation, hypoxia induced an increase in CTGF release from 49.8 ± 3.5 to 108.0 ± 13.7 pg/mL (n = 4) (Fig 1B). Next, we used GLI-1 siRNA and GLI-2 siRNA to investigate the role of GLI-1 and GLI-2 in hypoxia-induced CTGF expression. A Previous study indicated that GLI-1 siRNA (100 nM) or GLI-2 (100 nM) attenuated TCF4 expression in pancreatic cancer cells [32]. In the present study, transfection of WI-38 cells with GLI-1 siRNA (100 nM) and GLI-2 siRNA (100 nM), both GLI-1 and GLI-2 siRNA significantly and independently attenuated CTGF expression by 87.6 ± 15.7% and 69.8 ± 7.2% after undergoing hypoxia treatment for 6 h (Fig 1C). Moreover, to verify that GLI-1 and GLI-2 are involved in hypoxia-induced CTGF expression, the cells were transfected with a CTGF-luciferase plasmid. Fig 1D shows that the treatment of cells with hypoxia for 6 h increased CTGF-luciferase activity, which was inhibited by the transfection of GLI-1 and GLI-2 siRNA. The cells transfected with GLI-1 siRNA (100 nM) and GLI-2 siRNA (100 nM) substantially inhibited hypoxia-induced CTGF-luciferase activity by 67 ± 21% and 73.8 ± 17%, respectively (Fig 1D). Next, the transfection of cells with GLI-1 (100 nM) and GLI-2 siRNA (100 nM) evidently and separately lowered GLI-1 and GLI-2 expression, ensuring that GLI-1 and GLI-2 siRNA were functional (Fig 1E). In addition, the transfection of NHLFs with GLI-1 siRNA (25 nM) and GLI-2 siRNA (25 nM) also inhibited hypoxia-induced CTGF expression by 64.5 ± 3.1% and 73.1 ± 9.8%, respectively (Fig 1F). These results revealed that GLI-1 and GLI-2 played major roles in hypoxia-induced CTGF expression in human lung fibroblasts.

### Hypoxia-Induced GLI-1 and GLI-2 Translocation

**Fig 2:**
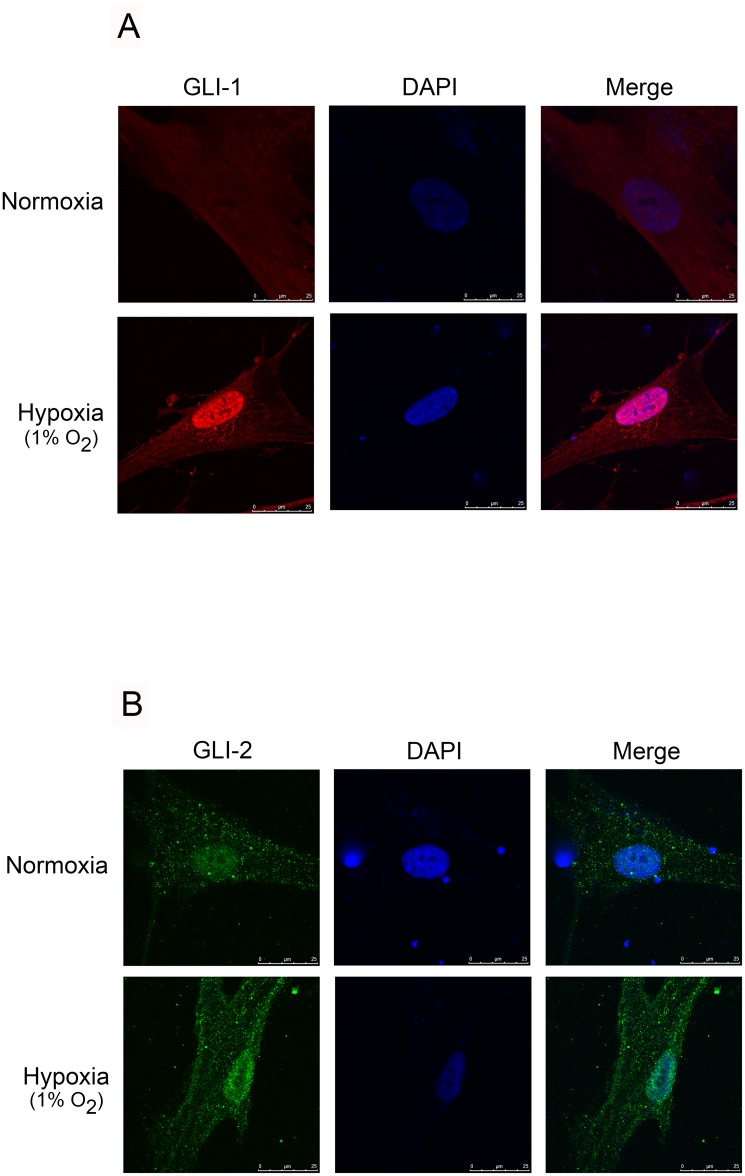
Hypoxia-induced translocation of GLI-1 and GLI-2 from the cytosol to the nucleus in WI-38 cells. A and B, WI-38 cells were subjected to hypoxia (1% O_2_) for 2 h. In confocal microscopy, the cells were incubated with antibodies specific for GLI-1 (A) and GLI-2 (B), and immunoreactivity was performed by incubating the cells with an FITC-conjugated secondary antibody. All slides were counterstained with DAPI (blue) to distinguish the nuclei, which were visualized under a fluorescence confocal microscope. The traces indicate similar results obtained from three independent experiments.

Aberger et al. (2012) reported that GLI-1 and GLI-2 translocate in the nucleus as the activators or co-activators of genes after a stimulus [12]. We performed immunofluorescence staining to examine these events, and found that GLI-1 and GLI-2 translocated from the cytosol into the nucleus after 2 h of hypoxia treatment (Fig 2A and 2B). These results revealed that hypoxia activated GLI-1 and GLI-2, which were involved in hypoxia-mediated CTGF expression in lung fibroblasts.

### MEK-, ERK-, and p38 MAPK-Mediated Hypoxia-Induced CTGF Expression

**Fig 3:**
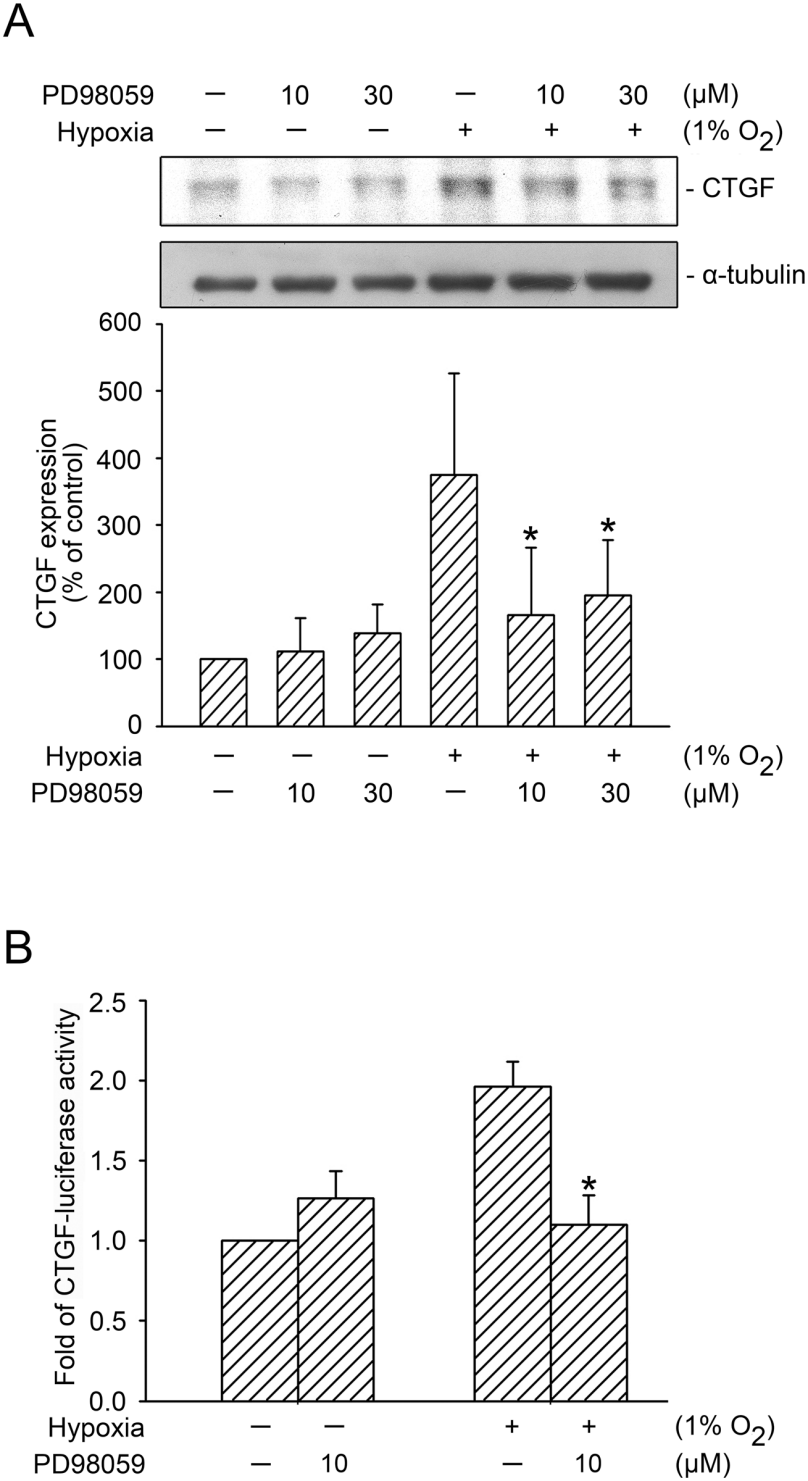
Involvement of MEK in hypoxia-induced CTGF expression. A, WI-38 cells were treated with PD98059 (an MEK inhibitor) for 20 min before they were subjected to hypoxia (1% O_2_) for an additional 6 h. CTGF and α-tubulin levels in cell lysates were detected using western blotting. The results are expressed as the mean ± SD of three independent experiments. * *p* < 0.05, compared with the hypoxia group without PD98059 treatment. B, WI-38 cells were transfected with 0.5 μg of CTGF-Luc and 0.1 μg of pBK-CMV-Lac Z for 24 h. The cells were treated with PD98059 before they were subjected to hypoxia (1% O_2_) for an additional 24 h. The luciferase activity assay is described in the “Materials and Methods” section. The results are expressed as the mean ± SEM of four independent experiments performed in duplicate. * *p* < 0.05, compared with the hypoxia group without PD98059 treatment.

**Fig 4:**
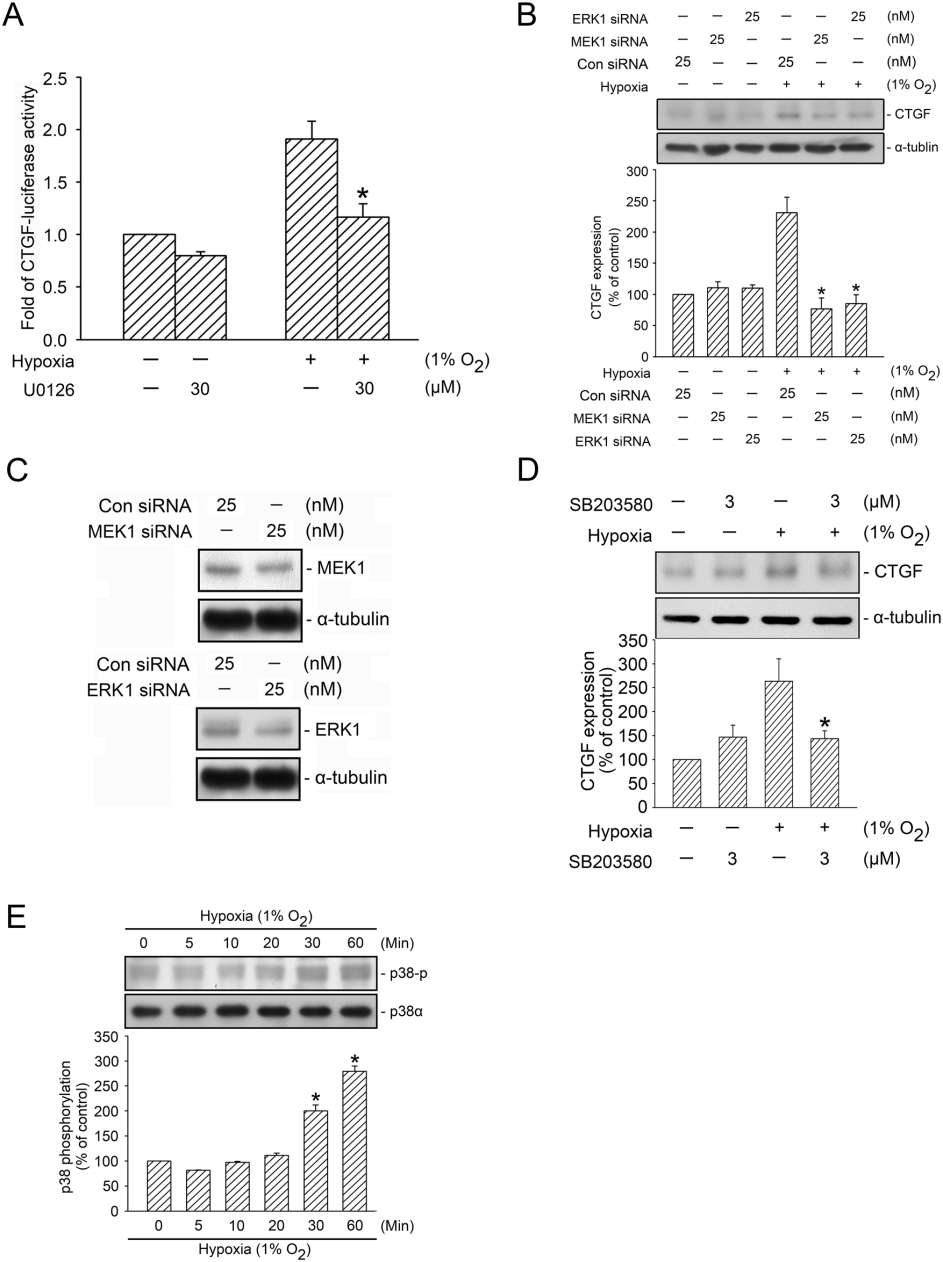
Involvement of ERK and p38 MAPK activation in hypoxia-induced CTGF expression. A, WI-38 cells were transfected with 0.5 μg of CTGF-Luc and 0.1 μg of pBK-CMV-Lac Z for 24 h. The cells were treated with U0126 before they were subjected to hypoxia (1% O_2_) for an additional 24 h. The luciferase activity assay is described in the “Material and Methods” section. The results are expressed as the mean ± SEM of three independent experiments performed in duplicate. * *p* < 0.05, compared with the hypoxia group without U0126 treatment. B, WI-38 cells were transfected with control siRNA (con siRNA), MEK1 siRNA, and ERK1 siRNA. After 24 h, the cells were subjected to hypoxia for an additional 24 h. CTGF and α-tubulin levels were detected using western blotting, as described previously. The results are expressed as the mean ± SEM of three independent experiments. * *p* < 0.05, compared with hypoxia plus the control siRNA group. C, WI-38 cells were transfected with control siRNA (con siRNA), MEK1 siRNA, and ERK1 siRNA. After 24 h of transfection, MEK1, ERK1, and α-tubulin levels were detected using western blotting. Typical traces represent three independent experiments that yield similar results. D, WI-38 cells were pretreated with SB203580 for 20 min before they were subjected to hypoxia (1% O_2_) for an additional 24 h. CTGF and α-tubulin levels in the cell lysates were detected using western blotting. The results are expressed as the mean ± SEM of five independent experiments. * *p* < 0.05, compared with the hypoxia group without SB203580 treatment. E, WI-38 cells were subjected to hypoxia (1% O_2_) for the indicated time intervals, after which the levels of p38 phosphorylation and p38α were detected using western blotting. The results are expressed as the mean ± SEM of three independent experiments. * *p* < 0.05, compared with the control group without hypoxia treatment.

Numerous studies have shown that GLI proteins are MEK/ERK substrates that are mediated by its activity [10, 23–25]. We used PD98059 (an MEK inhibitor) to investigate the role of MEK in GLI-1- and GLI-2-mediated hypoxia-induced CTGF expression in WI-38 cells. Treating cells with 10–30 μM of PD98059 inhibited hypoxia-induced CTGF expression, whereas treating cells with 10 μM of PD98059 reduced hypoxia-induced CTGF expression by 80.4 ± 26.9% (n = 4) (Fig 3A). Moreover, 10 μM of PD98059 completely inhibited the hypoxia-induced CTGF-luciferase activity (Fig 3B). A past study indicated that U0126 (an ERK inhibitor, 30 μM) inhibited curcumin-induced HO-1 expression in breast cancer cells [33]. Similarly, in the present study, the treatment of cells with U0126 (30 μM) inhibited hypoxia-induced CTGF-luciferase activity by 59.2 ± 13.5% (Fig 4A). We also used MEK1 siRNA (25 nM) and ERK1 siRNA (25 nM) as a specific knock down, and found that MEK1 siRNA and ERK1 siRNA both completely inhibited hypoxia-induced CTGF expression (Fig 4B). To ensure that MEK1 siRNA and ERK1 siRNA were functional, we transfected cells with MEK1 siRNA (25 nM) and ERK1 siRNA (25 nM), which respectively inhibited MEK1 and ERK1 expression (Fig 4C). Rong et al. (2005) found that p38 MAPK mediated hypoxia-induced CTGF expression in renal fibroblasts [34]. Therefore, we also examined whether p38 MAPK plays a role in hypoxia-induced CTGF expression in WI-38 cells. Hypoxia-induced CTGF expression decreased significantly in cells treated with 3 μM of SB203580 (a p38 MAPK inhibitor) (Fig 4D). Moreover, hypoxia induced an increase in p38 MAPK Tyr182 phosphorylation in a time-dependent manner, with a peak effect at 60 min (Fig 4E). These data revealed that MEK1, ERK1, and p38 MAPK were involved in hypoxia-induced CTGF expression. This study focused on the molecular mechanisms of the MEK1-dependent ERK signaling pathway in hypoxia-induced CTGF expression.

### ERK-Mediated Hypoxia-Induced GLI-1 and GLI-2 Translocation

**Fig 5:**
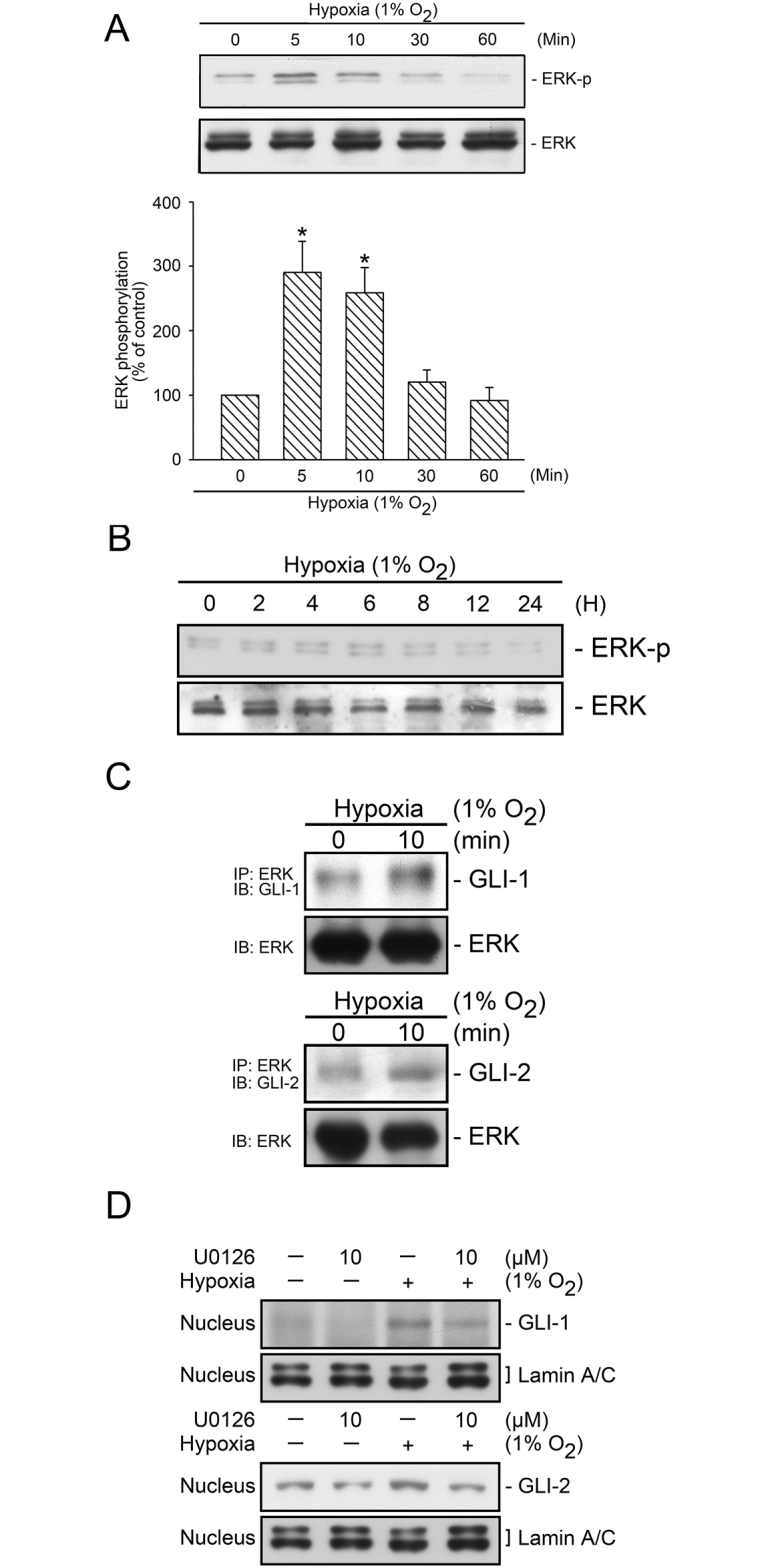
Involvement of ERK in hypoxia-induced GLI-1 and GLI-2 translocation. WI-38 Cells were subjected to hypoxia (1% O_2_) for a time interval of 0–60 min (A) or 2–24 h (B), after which the levels of ERK phosphorylation and ERK in cell lysates were detected using western blotting. The results are expressed as the mean ± SEM of three and four independent experiments. * *p* < 0.05, compared with the control group without hypoxia treatment. C, WI-38 cells were subjected to hypoxia (1% O_2_) for 10 min, before they were immunoprecipitated with a specific ERK antibody and mag sepharose magnetic beads, as described in the “Materials and methods” section. After elusion from the beads, the levels of GLI-1, GLI-2, and ERK in the lysates were detected using western blotting. Typical traces represent three independent experiments that yield similar results. D, WI-38 cells were pretreated with U0126 for 20 min before they were subjected to hypoxia (1% O_2_) for an additional 2 h, after which nuclear protein was collected. The levels of GLI-1, GLI-2, and lamin A/C in the nuclear extract were detected using western blotting. Typical traces represent three independent experiments that yield similar results.

We further evaluate whether hypoxia stimulates ERK activation. The Tyr204 phosphorylation causes an enzymatic stimulation in ERK [35]. Fig 5A shows that hypoxia time-dependently induced ERK Tyr204 phosphorylation, which peaked at 5 and 10 min and declined after 30 min of hypoxia stimulation (Fig 5A). We also attempted to determine whether ERK would be activated with prolonged hypoxia exposure. Fig 5B shows that in cells treated with hypoxia for 2–24 h, hypoxia did not induce further ERK Tyr204 phosphorylation (Fig 5B). These data implied that ERK plays a role in acute hypoxia stimulation. To identify the association between ERK and GLI proteins, we performed coimmunoprecipitation and nuclear protein extraction. The treatment of cells with hypoxia led to an association with ERK, GLI-1 and ERK, GLI-2 (Fig 5C). Moreover, we found that U0126 (10 μM) inhibited hypoxia-induced GLI-1 and GLI-2 nucleus translocation (Fig 5D). These results revealed that hypoxia activated ERK, which in turn mediated hypoxia-induced GLI-1 and GLI-2 nucleus translocation.

### Involvement of MEKK1 in Hypoxia-Induced CTGF Expression

**Fig 6:**
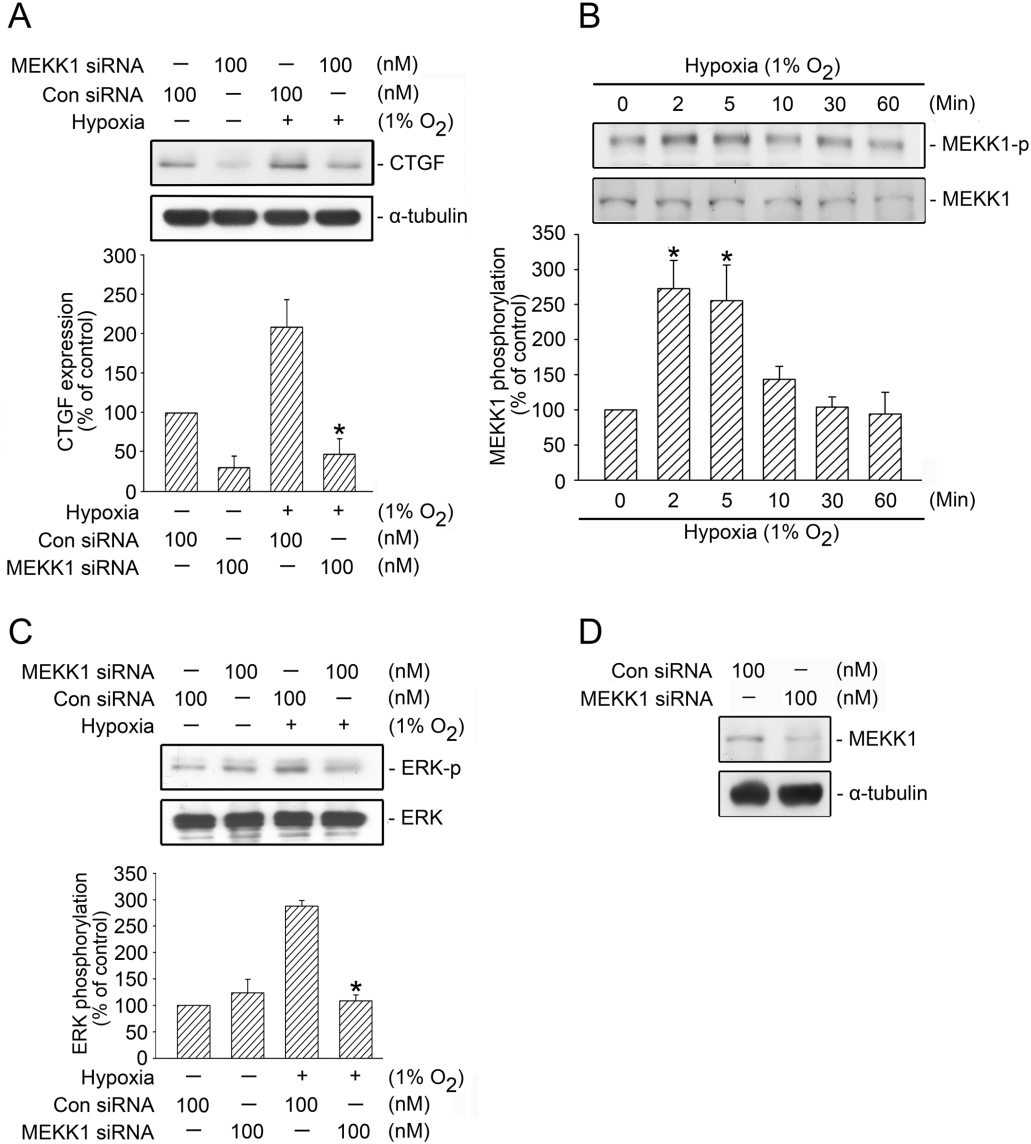
Involvement of MEKK1 in hypoxia-induced ERK activation and CTGF expression in WI-38 cells. A, WI-38 cells were transfected with control siRNA (con siRNA) or MEKK1 siRNA for 24 h before they were subjected to hypoxia (1% O_2_) for an additional 6 h. The CTGF and α-tubulin levels in the cell lysates were detected using western blotting. The results are expressed as the mean ± SEM of three independent experiments. * *p* < 0.05, compared with hypoxia plus the control siRNA group. B, WI-38 cells were subjected to hypoxia (1% O_2_) for 0–60 min. The levels of MEKK1 phosphorylation and MEKK1 expression in the cell lysates were detected using western blotting. The results are expressed as the mean ± SEM of three independent experiments. * *p* < 0.05, compared with the control group without hypoxia treatment. C, WI-38 cells were transfected with control siRNA (con siRNA) or MEKK1 siRNA for 24 h before they were subjected to hypoxia (1% O_2_) for an additional 5 min. The levels of ERK phosphorylation and ERK expression in the cell lysates were detected using western blotting. The results are expressed as the mean ± SEM of three independent experiments. * *p* < 0.05, compared with hypoxia plus the control siRNA group. D, Cells were transfected with MEKK1 siRNA for 24 h. The MEKK1 and α-tubulin levels in the cell lysates were detected using western blotting. The typical traces display similar results obtained from three independent experiments.

ERK is a downstream molecule of MEKK1 that regulates ERK activity [36]. To examine whether MEKK1 participates in hypoxia-induced CTGF expression, we used MEKK1 siRNA, 100 nM of which attenuated hypoxia-induced CTGF expression by 84.3 ± 18% (Fig 6A). The threonine phosphorylation of residue 261 in MEKK1 results in enzymatic activation [37]. We found that hypoxia time-dependently induced MEKK1 Thr261 phosphorylation, which peaked at 2 and 5 min and declined after 10 min of hypoxia treatment (Fig 6B). These data indicated that hypoxia activated MEKK1 and participated in hypoxia-induced CTGF expression. We also investigated the role of MEKK1-mediated hypoxia-induced ERK activation and found that MEKK1 siRNA (100 nM) completely inhibited hypoxia-induced ERK Tyr-204 phosphorylation (Fig 6C). To ensure that MEKK1 siRNA was functional, we transfected cells with MEKK1 siRNA (100 nM), after which MEKK1 expression was attenuated (Fig 6D). Overall, these results implied that hypoxia activated MEKK1 and participated in ERK-mediated hypoxia-induced CTGF expression.

### Contribution of AP-1 to Hypoxia-Induced CTGF Expression

**Fig 7:**
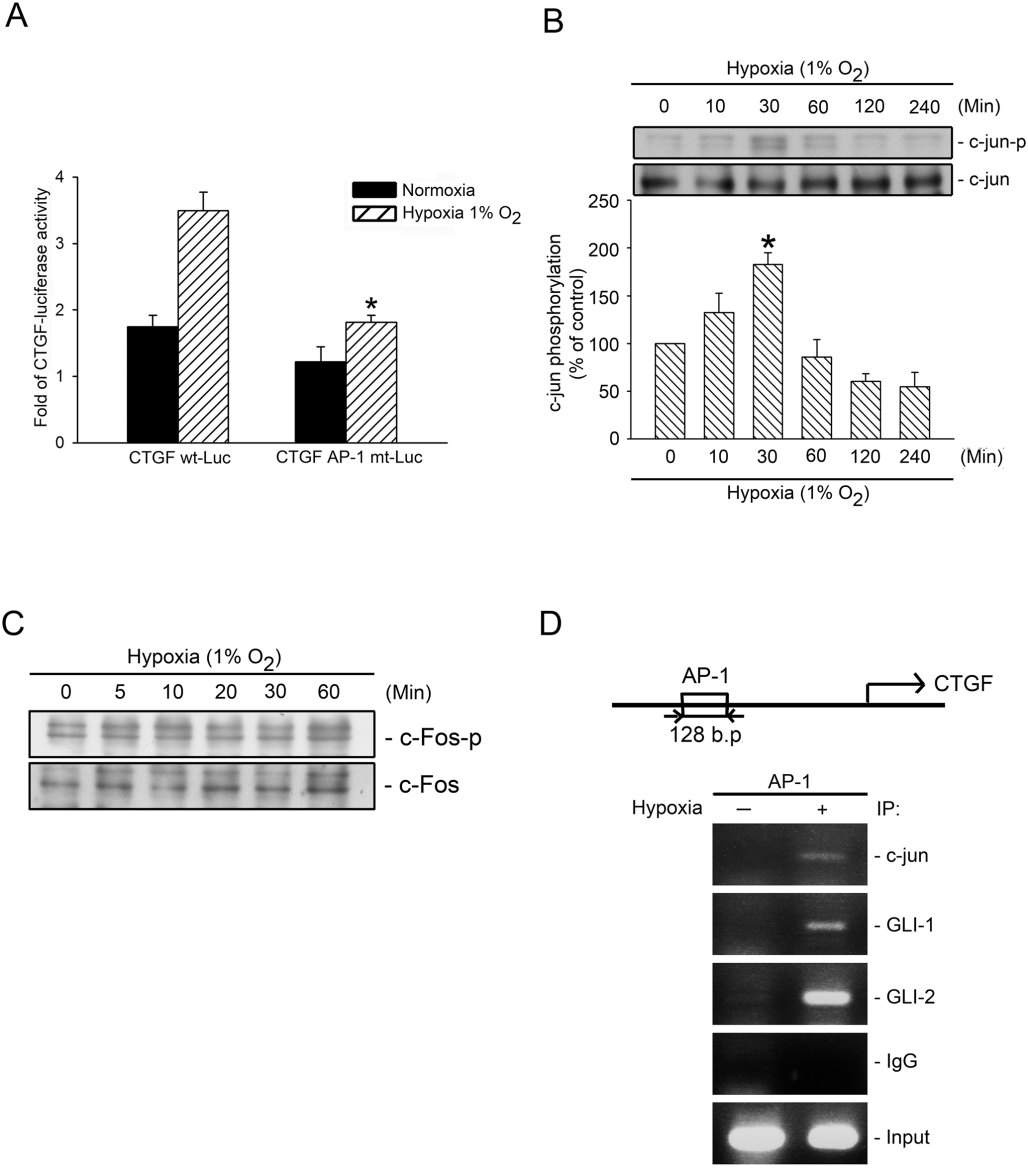
Involvement of AP-1 in hypoxia-induced CTGF expression. A, WI-38 cells were transfected with 0.5 μg of CTGF wt-Luc, 0.5 μg of AP-1-mt-CTGF-Luc (CTGF AP-1 mt-Luc), and 0.1 μg of pBK-CMV-Lac Z for 24 h after which they were subjected to hypoxia (1% O_2_) for an additional 24 h. The luciferase activity assay is described in the “Material and Methods” section. The results are expressed as the mean ± SEM of three independent experiments performed in duplicate. * *p* < 0.05, compared with hypoxia treatment with CTGF wt-Luc transfection. WI-38 Cells were subjected to hypoxia (1% O_2_) for the indicated time intervals, after which the levels of c-Jun phosphorylation and c-Jun (B) or c-Fos phosphorylation and c-Fos (C) in the cell lysates were detected using western blotting. The results are expressed as the mean ± SEM of three independent experiments. * *p* < 0.05, compared with the control group without hypoxia treatment. D, Incubated cells subjected to hypoxia (1% O_2_) for 2 h and detected using the ChIP assay, as described in the “Material and Methods” section. The typical traces indicate the three independent experiments that yield similar results.

Yu et al. (2009) found that the CTGF promoter contains several transcriptional factor binding sites (e.g., AP-1) [22]. To determine whether AP-1 plays a major role in CTGF expression after being subjected to hypoxia, we transfected CTGF-Luc and CTGF AP-1-mutated-Luc into WI-38 cells. Fig 7A shows that hypoxia-induced CTGF-luciferase activity decreased markedly by 65.8 ± 5.8% in cells transfected with a CTGF AP-1 mutated-Luc construct (Fig 7A). Both c-Jun and c-Fos are AP-1 subunits. To identify the AP-1 components that participate in GLI-1/-2-mediated hypoxia-induced CTGF expression, we tested c-Jun and c-Fos phosphorylation, and found that hypoxia induced an increase in c-Jun Ser63 phosphorylation, whereas hypoxia did not affect c-Fos Ser374 phosphorylation (Fig 7B and 7C). To further ascertain whether AP-1 plays a role in GLI-1/GLI-2-mediated hypoxia-induced CTGF expression, we performed ChIP to test whether c-Jun, GLI-1, and GLI-2 all bind to the CTGF promoter after exposure to hypoxia. The treatment of cells with hypoxia augmented the binding of c-Jun, GLI-1, and GLI-2 to the AP-1 binding site of the CTGF promoter region (Fig 7D). This result implied that GLI-1 and GLI-2 acted as coactivators with c-Jun in binding to the AP-1 binding site, and finally, mediating hypoxia-induced CTGF expression.

### GLI Regulation in Hypoxia-Induced α-SMA and Collagen Expression

**Fig 8:**
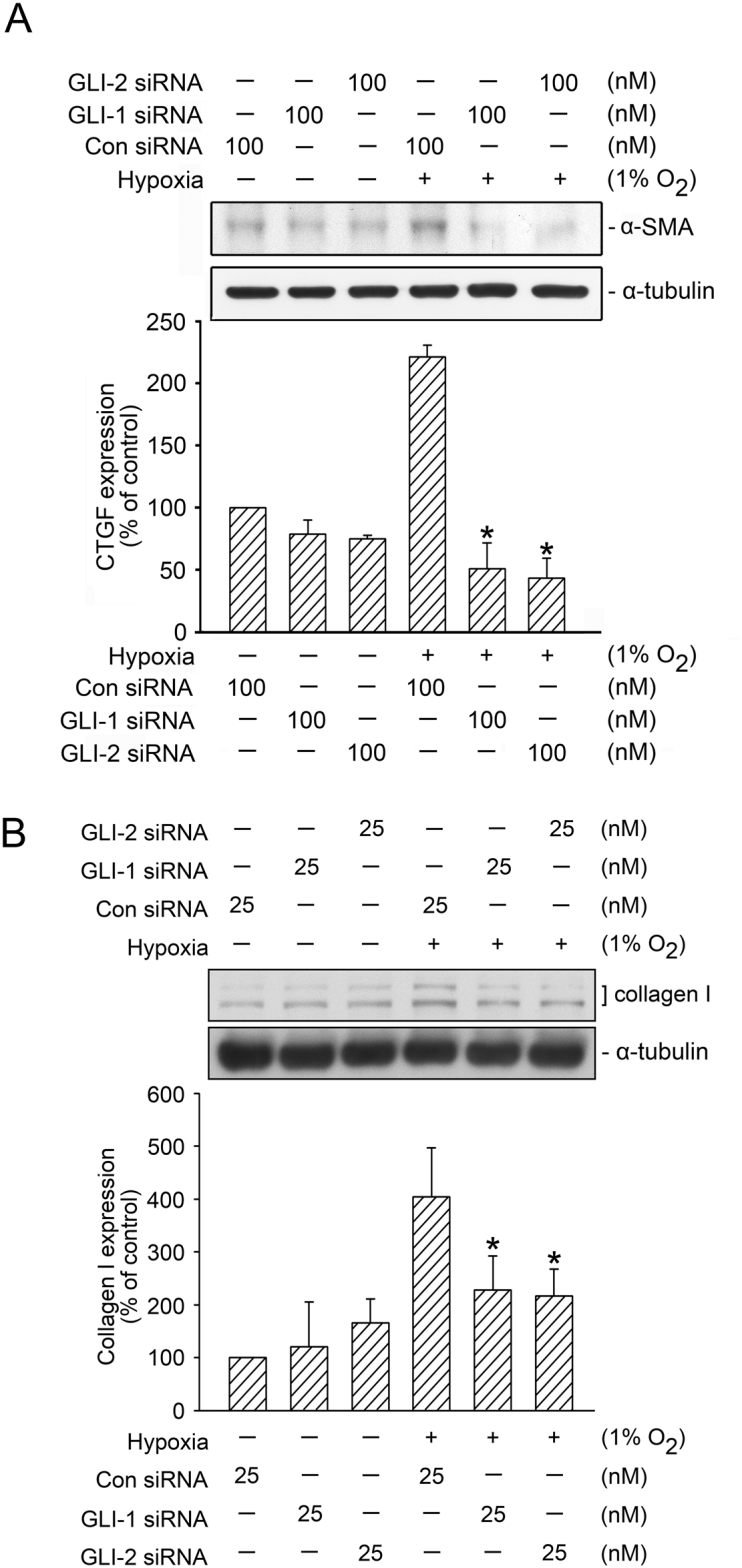
GLI-1 and GLI-2 mediated hypoxia-induced α-SMA and collagen expression. A, WI-38 cells were transfected with control siRNA (con siRNA), GLI-1 siRNA, and GLI-2 siRNA before they were subjected to hypoxia (1% O_2_) for an additional 24 h. The α-SMA and α-tubulin levels in cell lysates were detected using western blotting. The results are expressed as the mean ± SEM of three independent experiments. * *p* < 0.05, compared with hypoxia plus the control siRNA group. B, WI-38 cells were transfected with control siRNA (con siRNA), GLI-1 siRNA, and GLI-2 siRNA before they were subjected to hypoxia (1% O_2_) for an additional 24 h. Collagen and α-tubulin levels in the cell lysates were detected using western blotting. The results are expressed as the mean ± SD of three independent experiments. * *p* < 0.05, compared with hypoxia plus the control siRNA group.

Bolanos et al. (2012) found that GLI-1 and GLI-2 were highly expressed in fibroblasts in pulmonary fibrosis [13]. The overexpressions of α-SMA and collagen are the critical processes for the progression of pulmonary fibrosis [4, 13]. We thus analyzed whether GLI proteins mediated hypoxia-induced α-SMA and collagen expression. As displayed in Fig 8A, the transfection of cells with GLI-1 siRNA (100 nM) and GLI-2 siRNA (100 nM) resulted in a marked and total reduction in hypoxia-induced α-SMA expression (Fig 8A). In addition, the transfection of GLI-1 siRNA (25 nM) and GLI-2 siRNA (25 nM) attenuated hypoxia-induced collagen expression by 64.6 ± 21.0% and 83.3 ± 16.8%, respectively (Fig 8B). These results revealed that GLI-1 and GLI-2 regulated hypoxia-induced α-SMA and collagen expression in WI-38 cells.

## Discussion

Increased CTGF overexpression has been identified in fibrotic tissue and it is crucial in interstitial pulmonary fibrosis. Hypoxia also participates extensively in interstitial pulmonary fibrosis. Severe pulmonary fibrosis leads to reduced ventilation of the lungs and causes hypoxia, resulting in fibroblast proliferation and differentiation [6, 38–40]. Several reports have indicated the critical involvement of GLI-1 and GLI-2 in IPF [13, 41]. Moshai et al. (2014) found that knocking down the activity of GLI-1 and GLI-2 improved bleomycin-induced pulmonary fibrosis in mice [14]. Therefore, GLI-1 and GLI-2 play a critical role in interstitial pulmonary fibrosis. Previous studies have found that hypoxia contributed to tissue fibrosis, including in the liver, kidney, and lungs [42, 43]. Another study found that hypoxia mediated CTGF overexpression during fibroblast differentiation and fibrosis development [6]. Higgins et al. (2004) reported that hypoxia activated hypoxia-inducible factor-1α (HIF-1α), MEK1, and p38 MAPK, which contribute to tissue fibrosis [43]. In addition, numerous studies have indicated that acute hypoxia (0–72 h) enhanced fibroblast migration and the productions of collagen and CTGF [39, 40, 44, 45]. Previous studies have also shown that p38 MAPK and HIF-1α mediated hypoxia-induced CTGF expression in renal tubular cells and trophoblasts, respectively [31, 46]. Furthermore, a recent study reported that the G-protein estrogen receptor mediates hypoxia-induced CTGF and α-SMA expression in cancer-associated fibroblasts [47]. In present study, we found that MEKK1, MEK1, ERK, or p38 MAPK participated in acute hypoxia-induced CTGF expression in human lung fibroblasts. In addition, GLI-1 and GLI-2 mediated hypoxia-induced α-SMA and collagen expression. These results implied that these acute hypoxia-mediated factors may play a critical role in the CTGF, α-SMA, and collagen expression. Understanding the pathway that is involved in GLI-1- and GLI-2-mediated hypoxia-induced CTGF expression in human lung fibroblasts is crucial.

The hedgehog pathway and GLI proteins play a major role in cell proliferation and patterning, including fibrosis formation, which can be activated via noncanonical pathways (e.g., ERK signaling pathway) [48, 49]. Studies have found that activating the GLI proteins contributes substantially to interstitial pulmonary fibrosis [13, 14, 41]. Bolanos et al. (2012) reported that the GLI protein level was elevated in human lung fibrosis tissue [13]. Moreover, a study involving the use of a mouse model reported that treatment with GLI-1 siRNA and GLI-2 siRNA markedly inhibited the progression and severity of interstitial pulmonary fibrosis [14]. Wang et al. (2009) indicated that CTGF was a key protein in hypoxia-induced pulmonary tissue fibrosis [8]. However, whether GLI-1 and GLI-2 mediate hypoxia-induced CTGF expression has yet to be determined. In this study, the treatment of cells with GLI-1 siRNA and GLI-2 siRNA attenuated hypoxia-induced CTGF expression in WI-38 and NHLF cells. In a similar manner, GLI-1 siRNA and GLI-2 siRNA attenuated hypoxia-induced CTGF-luciferase activity. In addition, hypoxia induced GLI-1 and GLI-2 translocation from cytosol to the nucleus. Moreover, treatment with GLI-1 siRNA and GLI-2 siRNA also apparently inhibited the hypoxia-induced expression of α-SMA and collagen in WI-38 cells. Overall, our results implied that GLI-1 and GLI-2 participate in hypoxia-induced CTGF, α-SMA, and collagen expression in human lung fibroblasts.

Increasingly more data are showing that MEKK1/MEK1/ERK modulates inflammation in airway epithelial cells [50]. MEKK1 is involved in hypoxia-induced apoptosis in cardiac myocytes [51]. Several studies have found that ERK is necessary for stimulating transcription factors, including AP-1, and for mediating profibrotic gene expression [28, 52]. Oubrahim et al. (2013) reported that PD98059 inhibited *Pasteurella multocida* toxin-induced CTGF expression in mouse fibroblasts [53]. The findings of our recent study indicated that ERK participated in CXCL-12-induced CTGF expression in human lung fibroblasts [28]. In the present study, we confirmed the roles of MEKK1, MEK1, and ERK1 in hypoxia-induced CTGF expression. We found that MEKK1 siRNA, PD98059, MEK1 siRNA, U0126 (an ERK inhibitor), and ERK1 siRNA inhibited hypoxia-induced CTGF expression or CTGF-luciferase activity in WI-38 cells. Furthermore, the treatment of cells with 1% O_2_ (hypoxia) induced MEKK1 and ERK phosphorylation. MEKK1 siRNA transfection in cells attenuated hypoxia-induced ERK phosphorylation. Thus, MEKK1 activation was involved in ERK activation and hypoxia-induced CTGF expression in lung fibroblasts. Because ERK is an upstream effector of GLI proteins [12], we also analyzed whether ERK participated in hypoxia-mediated GLI-1 and GLI-2 activation. Moreover, we found that U0126 inhibited hypoxia-induced GLI-1 and GLI-2 translocation into the nucleus. The results of our coimmunoprecipitation experiments revealed that hypoxia induced ERK to become associated with GLI-1 or GLI-2. These data revealed that ERK was an upstream molecule of GLI-1 and GLI-2 through hypoxia stimulation. In addition to ERK, p38 MAPK has been indicated in several reports to participate in hypoxia-mediated pulmonary artery fibroblasts proliferation [54] and hypoxia-induced CTGF expression in human renal interstitial fibroblasts [34]. In the current study, we found that SB203580 (a p38 MAPK inhibitor) also attenuated hypoxia-induced CTGF expression. The stimulation of cells with hypoxia resulted in increase in p38 MAPK phosphorylation. All of these results showed that MEKK1/MEK1/ERK mediated hypoxia-induced GLI-1 and GLI-2 activation which is necessary for hypoxia-induced CTGF expression. Moreover, the current results indicated that p38 MAPK also participated in hypoxia-caused CTGF expression.

Several reports have indicated that AP-1 is crucial for regulating CTGF expression [22, 55]. In our previous studies, AP-1 was found to play a major role in CTGF expression through CXCL-12 and thrombin stimulation in human lung fibroblasts [22, 28]. In the current study, we found that treating cells with AP-1-mutated CTGF-luciferase attenuated hypoxia-induced CTGF-luciferase activity. In addition, our results revealed that hypoxia time-dependently induced an increase in c-Jun phosphorylation. However, the treatment of cells with hypoxia (1% O_2_) for 0–60 min did not affect c-Fos phosphorylation. These results implied that c-Jun, but not c-Fos, participates in hypoxia-induced CTGF expression. Because the CTGF promoter region does not contain a GLI-1/GLI-2-binding site, we tested whether GLI-1 and GLI-2 cooperated with c-Jun in binding to the AP-1-binding region and promoted CTGF expression. Our ChIP assay results revealed that hypoxia stimulation led to the direct binding of c-Jun, GLI-1, and GLI-2 to the AP-1 promoter region, which cooperated with GLI-1/GLI-2 and mediated hypoxia-induced CTGF expression.

The results of our study also implied that GLI-1 and GLI-2 play a crucial role in hypoxia-induced CTGF expression via the MEKK1/MEK1/ERK1 and AP-1 signaling pathways. However, using a fibroblast is not the only approach to simulating the mechanism of IPF. The roles of GLI-1 and GLI-2 in hypoxia-induced CTGF, α-SMA, and collagen expression require further investigation in other cell types (e.g., lung epithelial cells). Furthermore, p38 MAPK has been evidenced as a downstream kinase of hypoxia. However, further effort is required to determine the participation of p38 MAPK in hypoxia-induced α-SMA and collagen expression. In our previous study, CTGF was found to mediate CXCL12-induced α-SMA expression [28]. Nevertheless, we must verify whether CTGF also mediates hypoxia-induced α-SMA and collagen expression.

**Fig 9:**
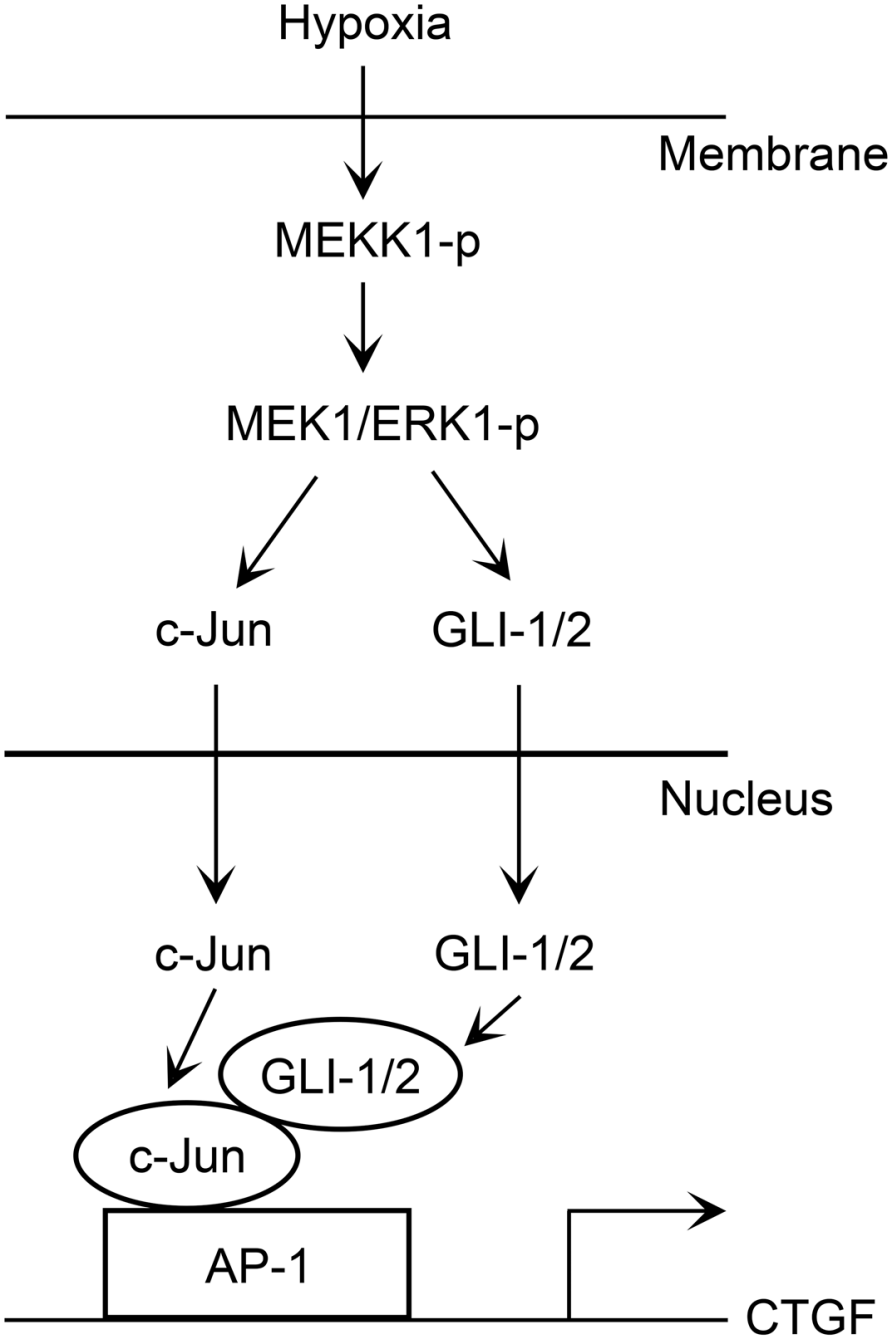
Simplified image displaying the results of hypoxia-induced CTGF expression mediated via the MEKK1/MEK1/ERK1/GLI-1/GLI-2 and AP-1 pathways in human lung fibroblasts. Hypoxia activates the MEKK1/MEK1/ERK1 pathways, which in turn activate GLI-1/GLI-2 inducing the translocation of both proteins from the cytosol to the nucleus, and then cooperating with AP-1 to induce CTGF expression. Moreover, both GLI-1 and GLI-2 mediate α-SMA and collagen expression through hypoxia stimulation in human lung fibroblasts.

In conclusion, we found that hypoxia activated the MEKK1/MEK1/ERK1/GLI-1/GLI-2 signaling pathway, after which it promoted GLI-1, GLI-2, and c-Jun binding to the AP-1 site of the CTGF promoter region to mediate CTGF expression in human lung fibroblasts. The p38 MAPK also participates in hypoxia-induced CTGF expression. Moreover, GLI-1 and GLI-2 mediate hypoxia-induced α-SMA and collagen expression. Fig 9 displays a simplified image of the pathway showing that hypoxia stimulates CTGF expression in lung fibroblasts via the MEKK1/MEK1/ERK1/GLI-1/GLI-2 and AP-1 pathways. Our findings detail a signal transduction pathway associated with GLI-1, GLI-2, CTGF, α-SMA, and collagen, and to explain how hypoxia induces CTGF expression.

## Abbreviations

AP-1: activator protein-1
ChIP: chromatin immunoprecipitation
CTGF: connective tissue growth factor
ECM: extracellular matrix
ERK: extracellular signal-regulated kinase
FGM: fibroblast growth medium
FITC: fluorescein isothiocyanate
GPER: G-protein estrogen receptor
HIF-1α: hypoxia-inducible factor-1α
ILD: interstitial lung disease
IPF: idiopathic pulmonary fibrosis
MAPK: mitogen-activated protein kinase
MEK: mitogen-activated kinase
MEKK1: MEK kinase 1
NHLF: normal human lung fibroblast
SD: standard deviation
SEM: standard error of mean
siRNA: small interfering RNA
α-SMA: α-smooth muscle actin
