# Correction: Induction of Connective Tissue Growth Factor Expression by Hypoxia in Human Lung Fibroblasts via the MEKK1/MEK1/ERK1/GLI-1/GLI-2 and AP-1 Pathways

**DOI:** 10.1371/journal.pone.0188608

**Published:** 2017-11-21

**Authors:** Yi Cheng, Chien-huang Lin, Jing-Yun Chen, Chien-Hua Li, Yu-Tin Liu, Bing-Chang Chen

The α-tubulin displayed in the bottom panel of Fig 4C is incorrect. Please see the complete correct Fig 4 here.

**Fig 4:**
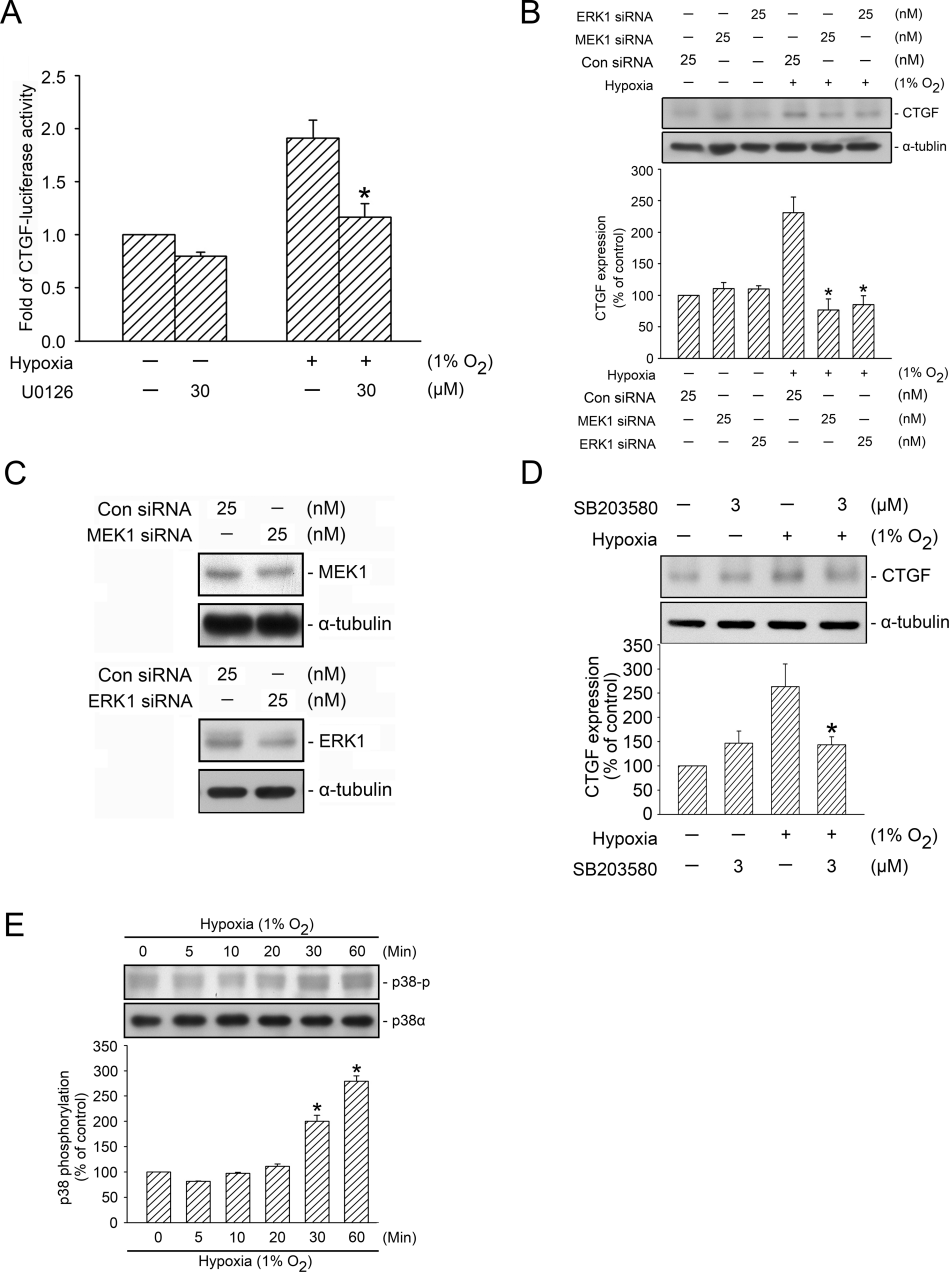
Involvement of ERK and p38 MAPK activation in hypoxia-induced CTGF expression. A, WI-38 cells were transfected with 0.5 μg of CTGF-Luc and 0.1 μg of pBK-CMV-Lac Z for 24 h. The cells were treated with U0126 before they were subjected to hypoxia (1% O_2_) for an additional 24 h. The luciferase activity assay is described in the “Material and Methods” section. The results are expressed as the mean ± SEM of three independent experiments performed in duplicate. ^*^ *p* < 0.05, compared with the hypoxia group without U0126 treatment. B, WI-38 cells were transfected with control siRNA (con siRNA), MEK1 siRNA, and ERK1 siRNA. After 24 h, the cells were subjected to hypoxia for an additional 24 h. CTGF and α-tubulin levels were detected using western blotting, as described previously. The results are expressed as the mean ± SEM of three independent experiments. ^*^ *p* < 0.05, compared with hypoxia plus the control siRNA group. C, WI-38 cells were transfected with control siRNA (con siRNA), MEK1 siRNA, and ERK1 siRNA. After 24 h of transfection, MEK1, ERK1, and α-tubulin levels were detected using western blotting. Typical traces represent three independent experiments that yield similar results. D, WI-38 cells were pretreated with SB203580 for 20 min before they were subjected to hypoxia (1% O_2_) for an additional 24 h. CTGF and α-tubulin levels in the cell lysates were detected using western blotting. The results are expressed as the mean ± SEM of five independent experiments. ^*^ *p* < 0.05, compared with the hypoxia group without SB203580 treatment. E, WI-38 cells were subjected to hypoxia (1% O_2_) for the indicated time intervals, after which the levels of p38 phosphorylation and p38α were detected using western blotting. The results are expressed as the mean ± SEM of three independent experiments. ^*^ *p* < 0.05, compared with the control group without hypoxia treatment.

In Fig 5D, the row containing Lamin A/C incorrectly appears twice. Please see the complete correct Fig 5 here.

**Fig 5:**
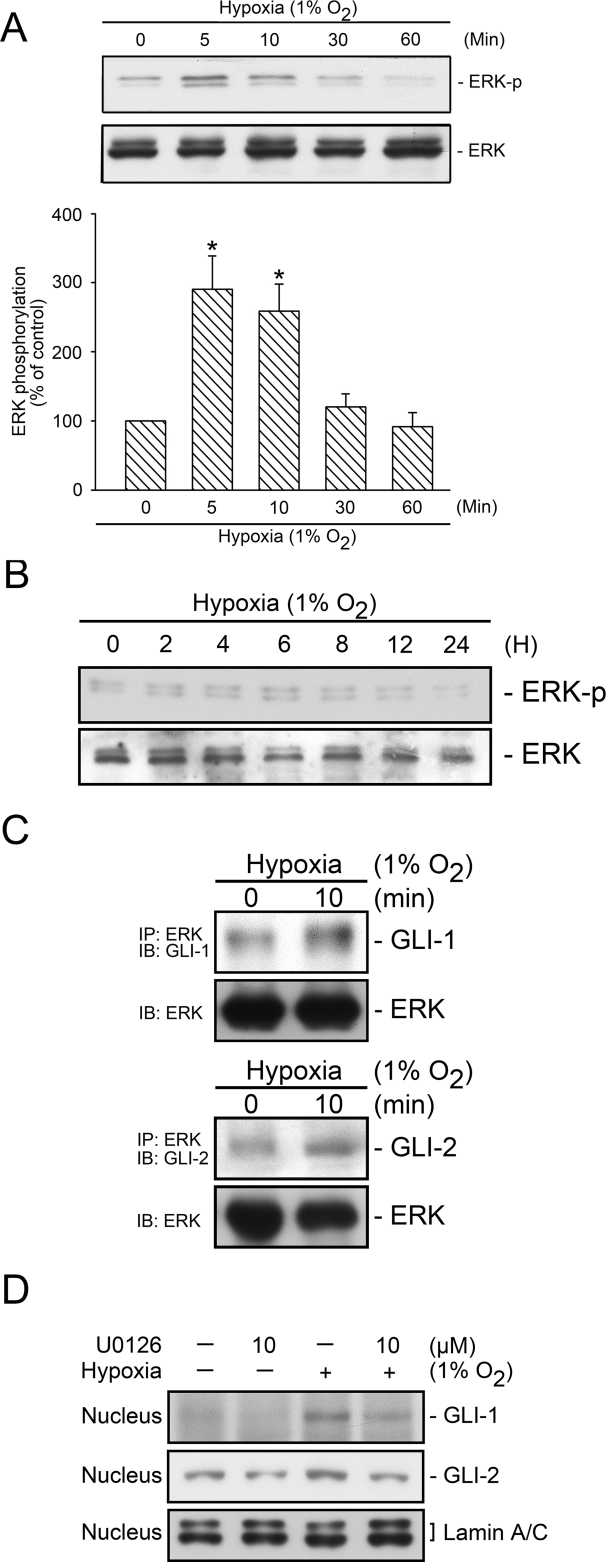
Involvement of ERK in hypoxia-induced GLI-1 and GLI-2 translocation. WI-38 Cells were subjected to hypoxia (1% O_2_) for a time interval of 0–60 min (A) or 2–24 h (B), after which the levels of ERK phosphorylation and ERK in cell lysates were detected using western blotting. The results are expressed as the mean ± SEM of three and four independent experiments. ^*^ *p* < 0.05, compared with the control group without hypoxia treatment. C, WI-38 cells were subjected to hypoxia (1% O_2_) for 10 min, before they were immunoprecipitated with a specific ERK antibody and mag sepharose magnetic beads, as described in the “Materials and methods” section. After elusion from the beads, the levels of GLI-1, GLI-2, and ERK in the lysates were detected using western blotting. Typical traces represent three independent experiments that yield similar results. D, WI-38 cells were pretreated with U0126 for 20 min before they were subjected to hypoxia (1% O_2_) for an additional 2 h, after which nuclear protein was collected. The levels of GLI-1, GLI-2, and lamin A/C in the nuclear extract were detected using western blotting. Typical traces represent three independent experiments that yield similar results.
